# Speed-Dependent Modulation of Muscle Activity Based on Muscle Synergies during Treadmill Walking

**DOI:** 10.3389/fnhum.2018.00004

**Published:** 2018-01-24

**Authors:** Benio Kibushi, Shota Hagio, Toshio Moritani, Motoki Kouzaki

**Affiliations:** ^1^Laboratory of Neurophysiology, Graduate School of Human and Environmental Studies, Kyoto University, Kyoto, Japan; ^2^Research Fellow of the Japan Society for the Promotion of Science, Tokyo, Japan; ^3^Graduate School of Education, The University of Tokyo, Tokyo, Japan; ^4^School of Health and Sport Sciences, Chukyo University, Nagoya, Japan

**Keywords:** electromyograms, motor control, center of activity, non-negative matrix factorization, central nervous system, joint acceleration

## Abstract

The regulation of walking speed is easily achieved. However, the central nervous system (CNS) must coordinate numerous muscles in order to achieve a smooth and continuous control of walking speed. To control walking speed appropriately, the CNS may need to utilize a simplified system for the control of numerous muscles. Previous studies have revealed that the CNS may control walking via muscle synergies that simplify the control of muscles by modularly organizing several muscles. We hypothesized that the CNS controls the walking speed by flexibly modulating activation of muscle synergies within one gait cycle. Then, we investigated how the activation of muscle synergies depend on walking speeds using the center of activity (CoA) that indicates the center of the distribution of activation timing within one gait cycle. Ten healthy men walked on a treadmill at 14 different walking speeds. We measured the surface electromyograms (EMGs) and kinematic data. Muscle synergies were extracted using non-negative matrix factorization. Then, we calculated the CoA of each muscle synergy. We observed that the CoA of each specific synergy would shift as the walking speed changed. The CoA that was mainly activated during the heel contact phase (C_1_) and the activation that contributed to the double support phase (C_3_) shifted to the earlier phase as the walking speed increased, whereas the CoA that produced swing initiation motion (C_4_) and the activation that related to the late-swing phase (C_5_) shifted to the later phase. This shifting of the CoA indicates that the CNS controls intensive activation of muscle synergies during the regulation of walking speed. In addition, shifting the CoA might be associated with changes in kinematics or kinetics depending on the walking speed. We concluded that the CNS flexibly controls the activation of muscle synergies in regulation of walking speed.

## Introduction

**Figure 1:**
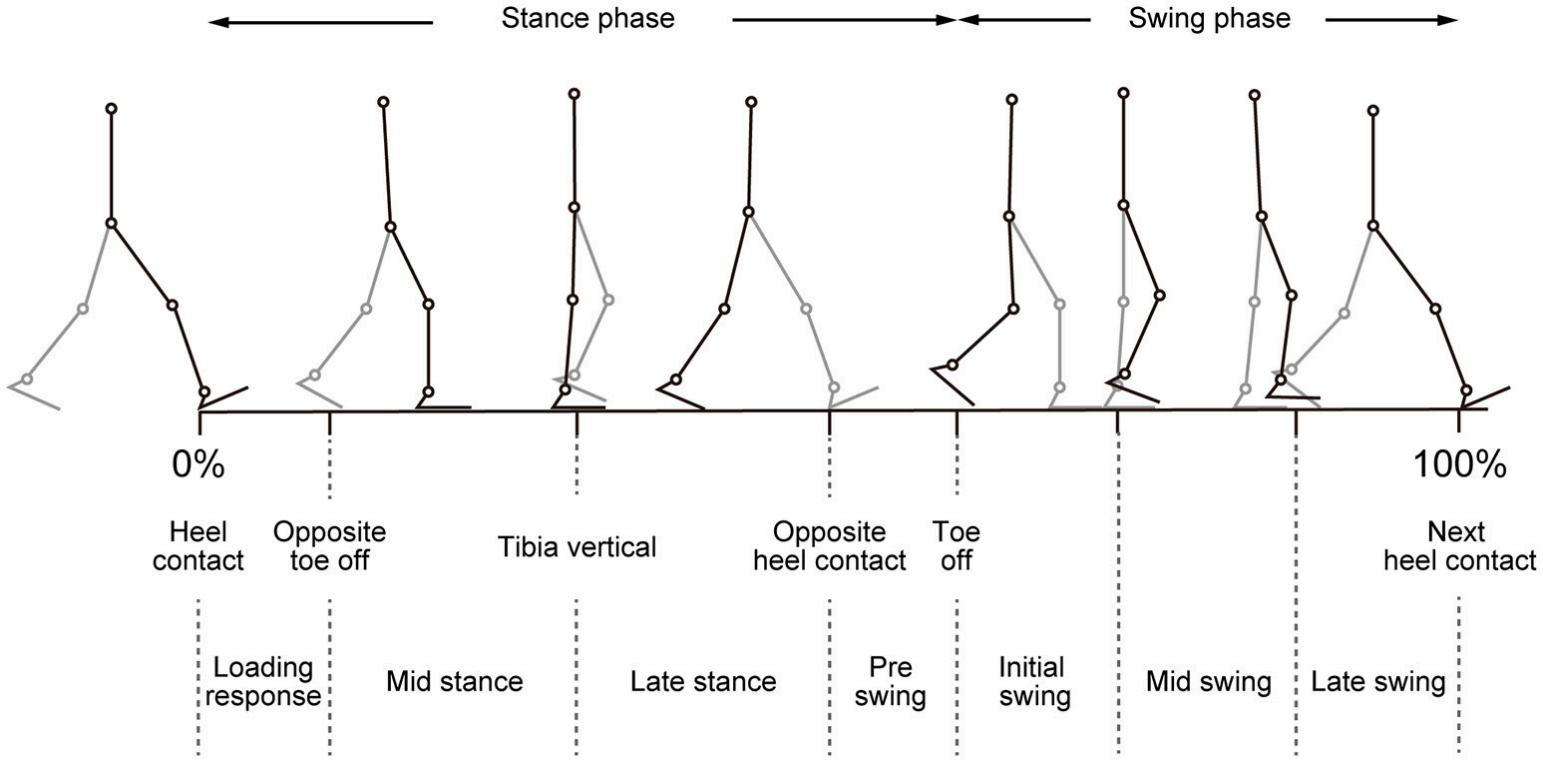
Illustration of walking phases. Different walking phases are shown with stick-pictures. In this figure, one gait cycle is defined from right heel contact to the next right heel contact. Right legs are colored black, and left legs are colored gray.

Healthy adults can easily adjust their walking speed in accordance with a given situation. Walking speed affects the kinematics and kinetics of walking. Detailed evidence of how the walking speed affects the kinematics and kinetics of walking can be found in energy consumption, join moment or muscle activities. Regarding the kinematics of the whole body, it has been revealed that energy consumption is most efficient at a usual or self-selected walking speed (Cavagna et al., 1963, 1976; Waters et al., 1988). The kinetics at each joint is also influenced by the walking speed. Pires et al. (2014) reported that the peak ankle plantar flexion moment for the propulsion phase and hip flexion moment for swing initiation increases as the walking speed increases (different walking phases are described in Figure 1). The work ratio is not positively correlated with the walking speed at the ankle joint, whereas the work ratio is positively correlated with the walking speed at the hip and knee joint (Chen et al., 1997). Thus, walking speed is a notable factor in identifying the mechanisms that control walking. To control walking speed, muscle activities also change when walking speed either increases or decreases. The amplitude of lower limb muscle activity tends to increase with the speed of progression (den Otter et al., 2004). Specifically, the peak root mean square of the tibialis anterior and medial gastrocnemius muscle activities linearly increases as walking speed increases (Warren et al., 2004). However, some muscle activities change non-linearly with an increasing walking speed as shown in the following literature. Murray et al. (1984) reported that the peak burst of gluteus medius muscle activity in the mid- or late-stance phase at slow walking speeds was larger than at fast walking speeds. In addition, the contribution of muscles for achieving walking subtasks depend on the walking speed. A detailed description follows below; the peak amplitude of hip extensor muscle activity in the mid-stance phase at extremely slow walking speeds is larger than at normal walking speeds (den Otter et al., 2004). This finding suggests that the muscle activity involved in single leg support largely contributes to slow walking speeds. Walking speed influences not only the amplitude of muscle activity but also the peak activation timing. In instances of fast walking speeds, gastrocnemius activity during the propulsion phase occurred earlier than at slow walking speeds (Murley et al., 2014). Thus, the muscle activity changes non-linearly as the walking speed increases or decreases. Because the human body includes numerous muscles, the musculoskeletal system has redundant degrees of freedom. The central nervous system (CNS) needs to construct a control system that solves the redundant degrees of freedom during walking.

To control a musculoskeletal system that has redundant degrees of freedom, the CNS must coordinate numerous muscles of the musculoskeletal system (Bernstein, 1967). It has been revealed that the main features of muscle activity patterns could be described by a few underlying components (Davis and Vaughan, 1993; Olree and Vaughan, 1995; Ivanenko et al., 2004). As a building block of low-dimensional control, the concept of muscle synergy, which simplifies the control of muscles by modularly organizing several muscles, has been proposed (Tresch et al., 1999; d'Avella et al., 2003; Hagio and Kouzaki, 2014; Hagio et al., 2015; Kubo et al., 2017; Nishida et al., 2017). In human walking, it has been revealed that sets of extracted muscle synergies characterize muscle patterns of specific phases of the gait cycle (e.g., the loading response phase, late stance phase or the swing phase) (Clark et al., 2010; Monaco et al., 2010; Barroso et al., 2014; Walter et al., 2014; Bejarano et al., 2017; Nazifi et al., 2017). A simulation study demonstrated that the combination of muscle synergies could allow flexible performance of walking subtasks, such as body support, forward propulsion and swing leg (Neptune et al., 2009). In addition to walking, muscle synergies are recruited during running, sidestepping, backward walking, uphill walking and perturbed walking (Chvatal and Ting, 2013; Oliveira et al., 2013; Zelik et al., 2014; Nishida et al., 2017; Saito et al., 2018). Thus, muscle synergies contribute to various aspects of locomotion. The number of muscle synergies was small in individuals with cerebral palsy, Parkinson disease, post-stroke individuals and spinal cord injured patients (Clark et al., 2010; Rodriguez et al., 2013; Danner et al., 2015; Steele et al., 2015). These findings indicate that muscle synergies may reflect disorders in the CNS. Therefore, the clinician may be able to evaluate walking ability by investigating muscle synergies. However, kinematics and kinetics of walking in people with certain health conditions (e.g., cerebral palsy, Parkinson disease, and hemiparesis) is different from that of healthy people (Balasubramanian et al., 2007; Rodriguez et al., 2013; Martino et al., 2014). Not only disorders in the CNS but also differences in kinematics or kinetics that are derived from disease may affect muscle synergies. In particular, walking speed is frequently reduced in individuals with disorders of the CNS (Balasubramanian et al., 2007; Clark et al., 2010; Steele et al., 2015). If muscle synergies depend on the walking speed, the clinicians may need to consider whether an observed difference of muscle synergy is derived from an effect of walking speed or disorders in the CNS. Therefore, investigating how muscle synergies in healthy people depend on the walking speed may contribute to the evaluation of walking ability in people with health conditions.

Because the construction of muscle synergies during walking is similar across different walking speeds (Ivanenko et al., 2004; Monaco et al., 2010), regulation of activation may contribute to controlling the walking speed. This suggestion is based on the finding that activation of muscle synergies can be altered by walking speed, the walk-to-run transition and the control of foot strike patterns (Hagio et al., 2015; Yokoyama et al., 2016; Nishida et al., 2017). Thus, the regulation of a locomotive task is influenced by the activation of muscle synergy. In addition, Chvatal and Ting (2013) reported that some muscle synergies are shared between posture control and walking, suggesting that the patterns of recruitment of muscle synergies may be individually controlled by each task. Because the kinetics and kinematics changed at different walking speeds, slow walking or fast walking is a different task under walking control. We supposed that the activation of muscle synergies during walking might change when the walking speed increases or decreases. Previous studies indicated that the fluctuation of the peak activation timing of modules was small at different walking speeds (Ivanenko et al., 2004; Cappellini et al., 2006). Therefore, the peak activation timing may not depend on the walking speed. We remarked in the previous paragraph that the kinematics of walking depend on the walking speed. The muscles' function or contribution to subtasks (e.g., body support, forward propulsion and leg swing) changes when walking speed decreases or increases (Liu et al., 2008; Neptune et al., 2008). Therefore, the activation of muscle synergies within one gait cycle may change with the progression of walking speed. To investigate changes in the activation of muscle synergies, we quantified the center of activity (CoA) that indicates the center of the distribution of activation timing within one gait cycle. We showed how the CNS controls the activation of muscle synergies with changing walking speeds by analysis of the CoA. In this study, our purpose was to identify how the CNS controls the walking speed based on the CoA.

## Methods

### Subjects

Ten healthy men (age: 23.3 ± 0.9; height: 171.1 ± 3.44 cm; weight: 64.1 ± 0.63 kg) participated in this study. The subjects provided written informed consent to participate in the study after receiving a detailed explanation of the purpose, potential benefits, and risks associated with participation. The experimental procedures were conducted in accordance with the Declaration of Helsinki and were approved by the Local Ethics Committee of the Graduate School of Human and Environmental Studies, Kyoto University (Approval number 26-H-22).

### Experimental setup and data procedures

Subjects walked on a treadmill (Adventure 3 PLUS, Horizon, Johnson Health Tech Japan Co., Tokyo, Japan) at 14 different controlled speeds (2.0, 2.5, 3.0, 3.5, 4.0, 4.5, 5.0, 5.5, 6.0, 6.5, 7.0, 7.5, and 8.0 km/h and a preferred walking speed of 4.3 ± 0.63 km/h) that were administered in a random order over the span of 50 gait cycles. To determine the preferred walking speed for each individual subject, we modulated the treadmill speed without showing the walking speed to the subjects. The preferred walking speeds were determined at the moment when the subjects felt comfortable. Surface electromyograms (EMG) were recorded from 12 muscles in the right lower limb and trunk: gastrocnemius medialis (MG), gastrocnemius lateralis (LG), soleus (SOL), tibialis anterior (TA), vastus lateralis (VL), rectus femoris (RF), biceps femoris (BF), tensor fasciae latae (TFL), adductor longus (AL), gluteus medius (Gmed), gluteus maximus (Gmax) and erector spinae (ERE). The electrode placement was carefully chosen by using an ultrasonic device to minimize crosstalk from the adjacent muscles, and we placed the EMG electrodes based on suggestions from SENIAM (seniam.org), the European project on surface EMGs. The EMG signals were amplified (SX230-1000, Biometrics, Gwent, UK) and bandpass filtered between 20 and 450 Hz (Kouzaki and Shinohara, 2010). All electrical signals were stored at a sampling frequency of 1,000 Hz on the hard disk of a personal computer using a 16-bit analog-to-digital converter (PowerLab/16SP; AD Instruments, Sydney, Australia). Kinematic data were recorded by using a 3D optical motion capture system with 12 cameras operating at 100 Hz (Optotrak®, Northern Digital Inc., Waterloo, Ontario). This system captured three-dimensional coordinates of reflective markers that were attached to anatomical landmarks on the subjects. The reflective markers that were attached to the subjects were positioned at the top, right and left side of their heads, as well as on the acromions, elbows, wrists, anterior superior iliac spine, posterior superior iliac spine, greater trochanters, medial and lateral epicondyles, medial and lateral malleolus, heels and toes. The measured reflective marker data were low-pass filtered at 5 Hz (Sawers et al., 2017). The subjects wore shoes that were equipped with foot switches to record heel contact. We defined one gait cycle as the time between initial right heel contact to the moment before the next right heel contact.

### EMG procedures

We analyzed 30 gait cycles within the sampled EMGs and walking speed for each subject. It has been revealed that the kinematics of initial walking differs from that of steady walking (Mbourou et al., 2003; Bus and de Lange, 2005); therefore, we excluded the initial 10 gait cycles from analysis. To extract muscle synergies, EMGs were high-pass filtered at 40 Hz, full-wave rectified, low-pass filtered at 10 Hz, and time-interpolated over 100 points (Clark et al., 2010). EMGs after preprocessing provided a 12 muscle × 30 gait cycle-sized matrix for each subject. The EMG matrix was normalized by the peak activity in EMGs for all muscles and the standard deviation of each muscle to allow for unit variance (Torres-Oviedo and Ting, 2007; Hagio and Kouzaki, 2014).

### Extracting muscle synergies

We extracted muscle synergies using a non-negative matrix factorization (NMF) algorithm (Lee and Seung, 1999; Tresch et al., 1999). This method is used to minimize error by approximately decomposing a matrix into two non-negative matrixes. The muscle activation pattern for a given walking speed is represented by the following equation:

$$M=\sum_{i=1}^{N}W_{i}\;C_{i}+\varepsilon \;W_{i}\geq 0\;C_{i}\geq 0$$

where *W*_*i*_ specifies the muscles that are involved in the synergy *i* and their relative contributions and ε is the residual. Each component of *W*_*i*_ represents the contribution of one particular muscle to that muscle synergy, and an individual muscle may contribute to multiple muscle synergies. The composition of the muscle synergy does not change during a certain walking speed, but each muscle synergy is multiplied by a scalar activation coefficient (*C*_*i*_) that does change during a certain walking speed. The weighting of muscle synergies and activation coefficient matrixes are normalized such that the individual muscle weighting vector is a unit vector.

### Selection of the number of muscle synergies

**Figure 2:**
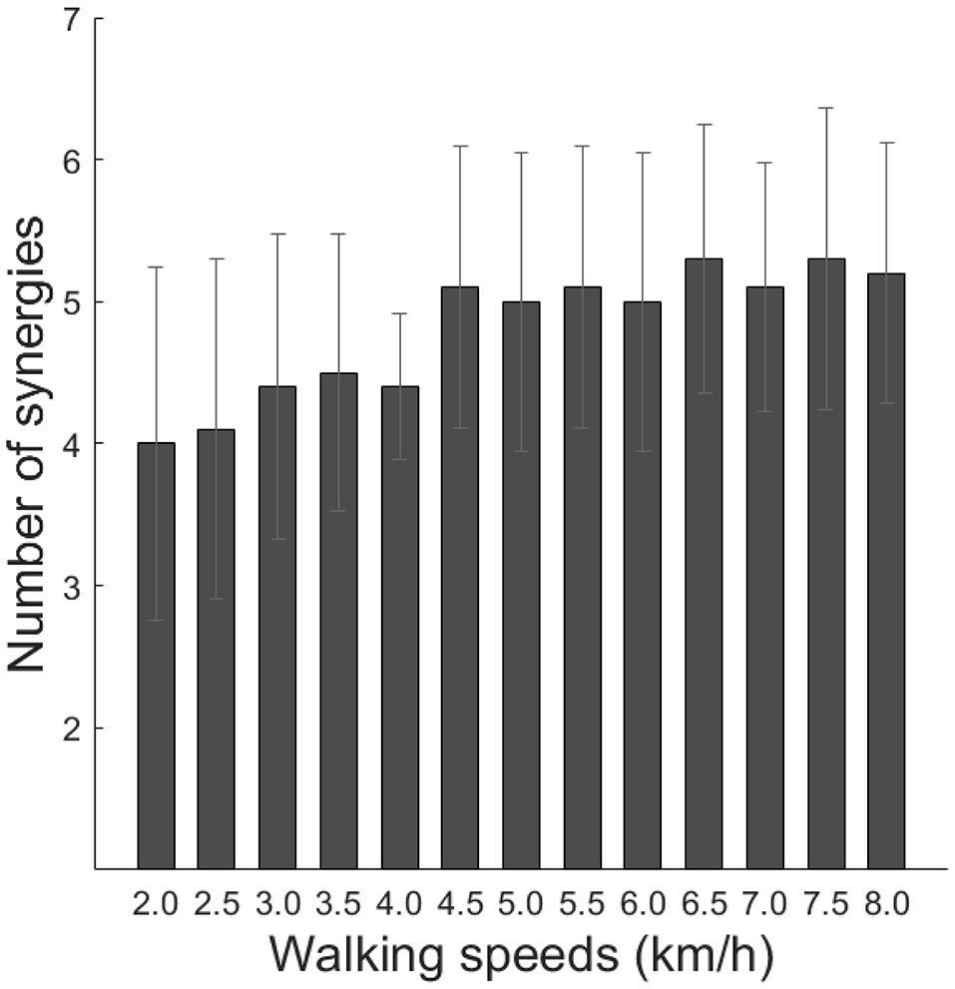
Number of synergies across walking speeds. Averaged number of synergies with cross-validation among subjects is indicated (black bars). Error bars denote the standard deviation.

We extracted between 1 and 12 muscle synergy matrixes and activation coefficient matrixes from the EMG data matrixes at each walking speed to select the number of synergies that could best model the EMG data, and these extractions were repeated 20 times for each subject (Cheung et al., 2005). We performed cross-validation to examine the consistency of the number of synergies. To perform cross-validation, we separated the EMG data (30 gait cycles) into 60% of the EMG (18 gait cycles) and 40% of the EMG (12 gait cycles) at each walking speed (Torres-Oviedo and Ting, 2007). We extracted muscle synergies from 60% of the EMG data and 40% of the EMG data. For updating the weightings and activations of 40% of the EMG data, activations of muscle synergies were updated, whereas the weightings of muscle synergies were fixed by weightings at 60% of the EMG (Cheung et al., 2005; Torres-Oviedo and Ting, 2007). This cross-validation was repeated 10 times. We verified the goodness of fit between the original and reconstructed EMG data matrixes to select the smallest number of synergies that resulted in an adequate reconstruction of the muscle responses. We calculated the variability accounted for (VAF) as a coefficient of determination, which was based on the entire dataset (global VAF) and each muscle (muscle VAF) for each subject (Torres-Oviedo et al., 2006; Clark et al., 2010; Hagio and Kouzaki, 2014). The VAF was calculated using the following equation:

$$VAF={\left(EMG_{o}-EMG_{r}\right)}^{2}/{EMG_{o}}^{2}\times 100$$

where *EMG*_*o*_ represents an original EMG data matrix, and *EMG*_*r*_ denotes a reconstructed EMG data matrix. The global VAF and 12-muscle VAF were calculated for 40% of the EMG. The global VAF matrix and the 12-muscle VAF matrixes were averaged among repetitions for each VAF matrix. In addition, the 95% confidence interval (CI) for the VAF matrix at each synergy number (1–12) was calculated. The number of synergies underlying each dataset was defined as the minimum number of synergies at which the lower bound of the 95% CI exceeded 90% global VAF and 75% muscle VAF (Clark et al., 2010; Hagio and Kouzaki, 2014; Sawers et al., 2015). The average number of synergies among subjects is illustrated in Figure 2. We rounded the median number of synergies and we determined that the number of synergies was 4 (2.0–4.0 km/h and preferred walking speeds) and 5 (4.5–8.0 km/h).

### Methodological identification

**Figure 3:**
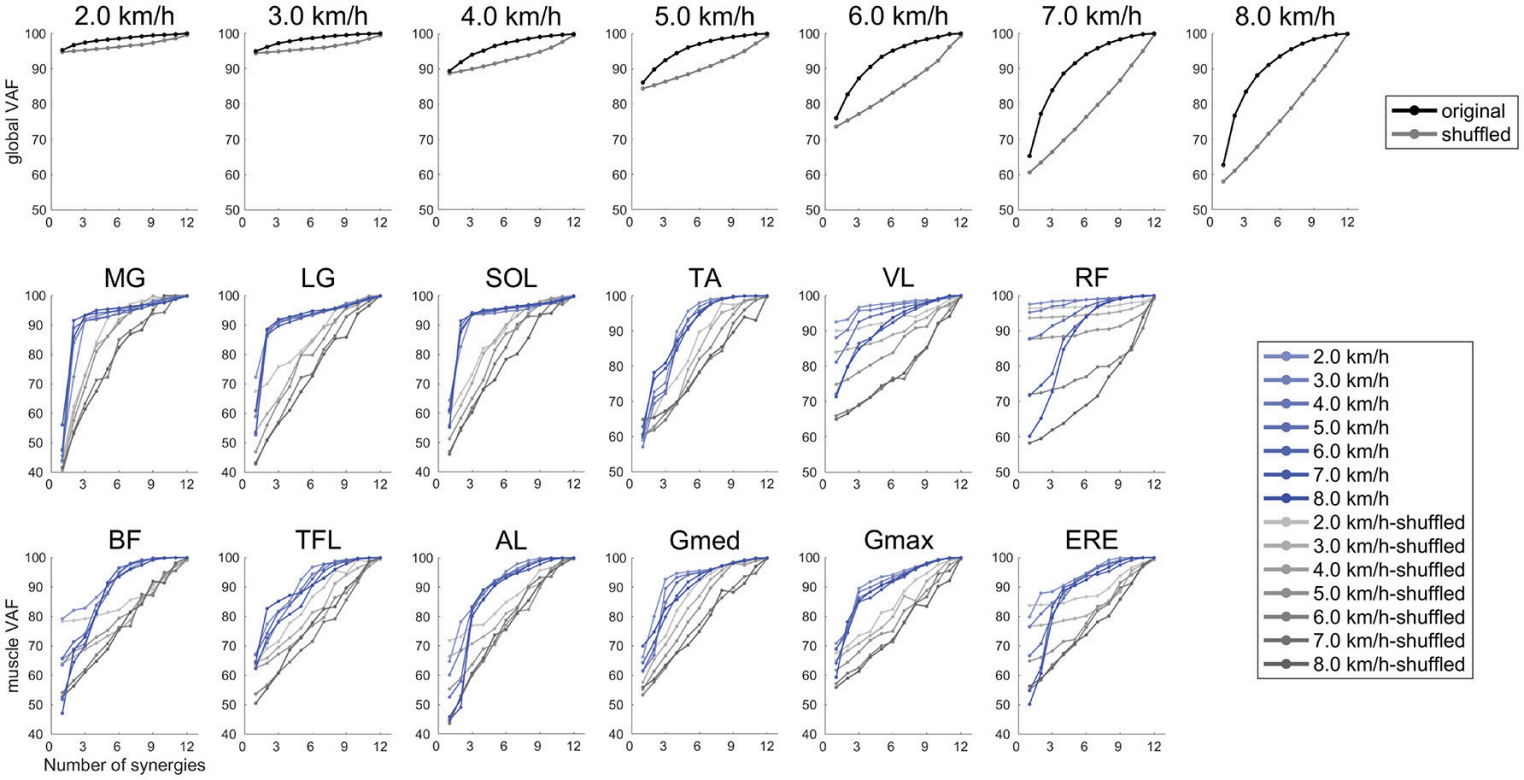
Results of global VAF and muscle VAF across walking speeds. Upper plots indicate the averaged global VAF (black) and shuffled VAF (gray) among the subjects. The middle and lower plots are averaged muscle VAF (blue) and shuffled VAF (gray) among the subjects.

To confirm that the muscle synergies extracted by the NMF algorithm were due to the inherent organization of muscle activation based on neurophysiological evidence rather than artifacts of the NMF method, the VAF levels were compared with the VAF values for muscle synergies that were extracted from shuffled datasets. For the shuffled procedure, the data for each muscle were shuffled independently; therefore, this shuffled data matrix contained the same values, range, and variance for each muscle, whereas the relationships among muscle activations were removed (Chvatal and Ting, 2013; Hagio and Kouzaki, 2014). Two types of averaged VAF (global VAF, muscle VAF) across walking speeds are shown in Figure 3. In all cases, the VAF values for the reconstruction of the original data using the identified muscle synergies were higher than the VAF for the shuffled datasets.

### Grouping of similar muscle synergies

We sorted muscle synergies by using cosine similarity (Hagio et al., 2015). Initial functional sorting was performed by grouping muscle synergies based on the values of cosine similarity to that of an arbitrary reference subject. The cosine similarity was calculated between reference muscle synergies and other muscle synergies. When the cosine similarities of W or C were over 0.71 (*p* < 0.01), muscle synergies were sorted as similar muscle synergies. If two muscle synergies at one walking speed were categorized into the same muscle synergy group, we defined the pair of muscle synergies with the highest correlation as the same group of muscle synergies (Torres-Oviedo and Ting, 2007; Hagio and Kouzaki, 2014). Subsequently, we computed an averaged set of similar muscle synergies for all subjects, and the similarity between the averaged muscle synergies and other muscle synergies was calculated.

### The center of activity in muscle synergies

We quantified the center of activity (CoA) as the center of the distribution of activation within one gait cycle. To quantify the CoA, we used circular statistics (Batschelet, 1981). The CoA among all subjects was calculated as the angle of the vector that pointed to the center of mass of that circular distribution (Martino et al., 2014, 2015; Sylos-Labini et al., 2014). We observed that peak activations were unclear at slow walking speeds. In that case, we could not analyze the data by shifting the peak timing in activations. To understand whether the center of the distribution of activation altered across different walking speeds, the CoA was selected as the assessment of activation. The CoA was quantified as the angle of the vector that pointed to the center of mass of that circular distribution. The CoA was calculated by using the following equations:

$$\begin{array}{l} A=\sum_{t=1}^{N}\left(cos\theta_{t}\times Act_{t}\right)\\ B=\sum_{t=1}^{N}\left(sin\theta_{t}\times Act_{t}\right)\\ \;CoA=tan^{-1}(B/A) \end{array}$$

where θ_*t*_ is an angle at a *t* point (1–100 points) that transformed the gait cycle (1–100%) into the angle θ (3.6–360°). *Act*_*t*_ is the activation amplitude of muscle synergy at *t*. The CoA is calculated in polar coordinates as the inverse tangent of B/A.

### Statistics

One-way analyses of variance were used to compare the CoA among walking speed conditions with *post hoc* comparisons between groups that included a Tukey-Kramer correction for multiple comparisons. Differences in the CoA were analyzed by using an unimpaired Student's *t*-test. Significance was set at *p* < 0.05 for all tests.

## Results

### Muscle activities

**Figure 4:**
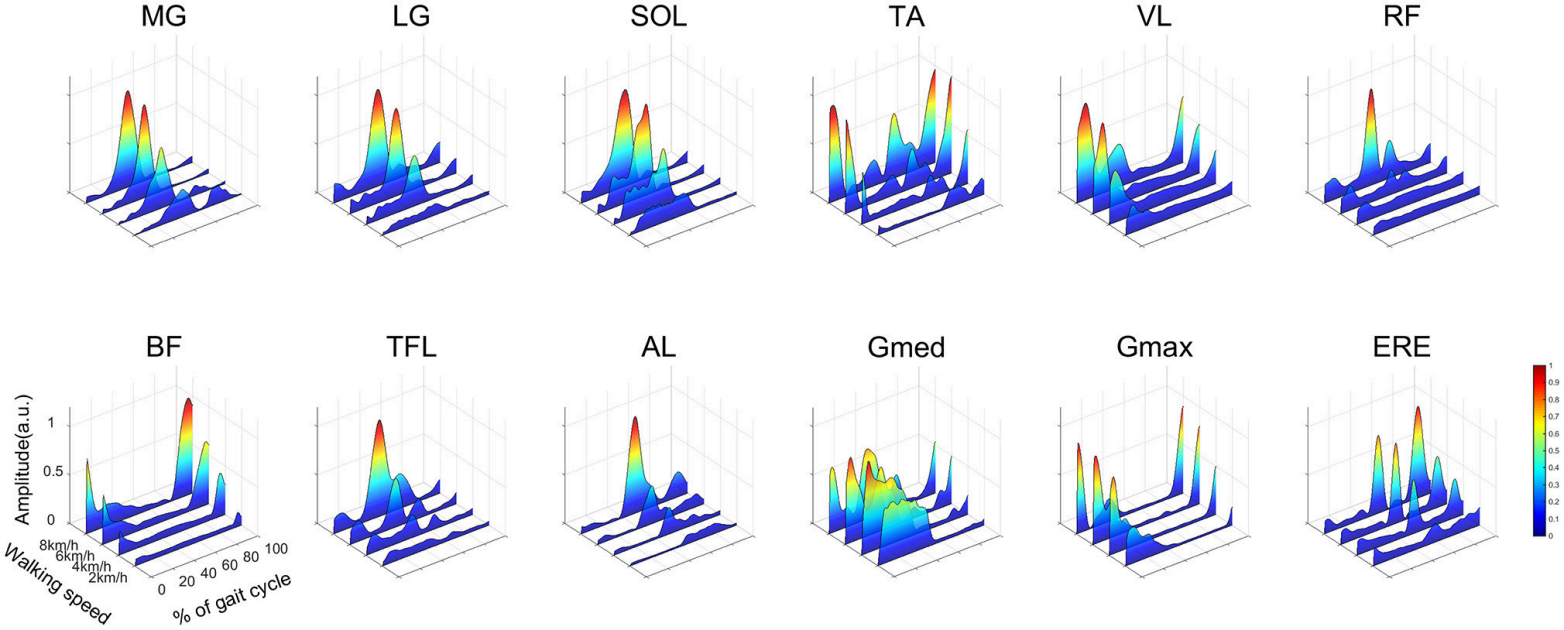
Muscle activities in a representative subject at 2.0, 4.0, 6.0, and 8.0 km/h. Each muscle activity was normalized by maximum activation among all walking speeds. Intensive muscle activity is illustrated by warm colors, and small muscle activity is illustrated by cold colors. The abbreviations denote the gastrocnemius medialis (MG), gastrocnemius lateralis (LG), soleus (SOL), tibialis anterior (TA), vastus lateralis (VL), rectus femoris (RF), biceps femoris (BF), tensor fasciae latae (TFL), adductor longus (AL), gluteus medius (Gmed), gluteus maximus (Gmax) and erector spinae (ERE).

The average representative EMGs at 2.0, 4.0, 6.0, and 8.0 km/h are illustrated in Figure 4. The ankle planter flexor muscles (MG, LG, SOL) mainly activated from the mid-stance phase to the late-stance phase, and peak activations during the propulsion phase were large at high walking speeds. The ankle dorsiflexor muscle (TA) activated between the late-swing phase and the heel contact phase. In addition, TA also activated during the mid-swing phase. The activation patterns of the knee extensors (VL) were similar to those of the hip extensors (BF, Gmax), and these muscles activated from the late-swing phase to the heel contact phase. The hip adductor muscle (Gmed) activated during the stance phase. The activation of the hip adductor muscle (Gmed) during the mid-stance phase decreased as the walking speed increased. Though the hip flexor muscle (RF) was mainly activated during the loading response, activations during the pre-swing phase were large when the walking speed increased.

### Extracted muscle synergies

**Figure 5:**
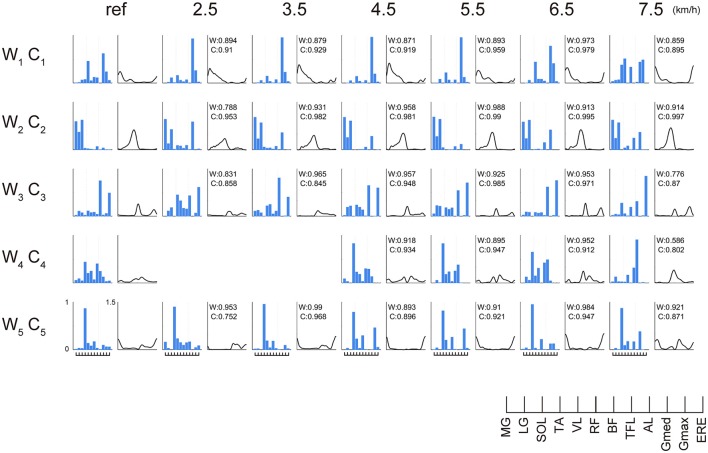
The weightings of muscle synergies and their activations across walking speeds in representative subjects. The muscle weightings (bar) and activations (line) are shown. To demonstrate muscle synergies in representative subjects, muscle synergies were sorted by comparing reference muscle synergies (average muscle synergies among subjects at 6.0 km/h) and the other muscle synergies in representative subjects. The values of cosine similarity between reference muscle synergies and the other muscle synergies are indicated in the upper side of the wave forms. Because we constrained the number of muscle synergies among subjects, variabilities of weightings and activations were relatively small. However, muscle synergies were slightly different among subjects. We considered that such a small difference might contribute to subject-specific walking. To evaluate characteristics during weighting or activation of muscle synergies per subject, we show a representative subject in Figure 5. The abbreviations indicate gastrocnemius medialis (MG), gastrocnemius lateralis (LG), soleus (SOL), tibialis anterior (TA), vastus lateralis (VL), rectus femoris (RF), biceps femoris (BF), tensor fasciae latae (TFL), adductor longus (AL), gluteus medius (Gmed), gluteus maximus (Gmax) and erector spinae (ERE).

Representative muscle synergies across walking speeds are illustrated in Figure 5. The muscle synergy W_1_ was mainly recruited between the heel contact phase and the single support phase, which distributed the knee extensor muscle (VL), hip extensor muscle (Gmax) and hip adductor muscle (Gmed). The W_1_ was related to the loading response and the single support. As the walking speed increased, the weighting of Gmed in the W_1_ decreased, and activations during the mid-stance phase were reduced. The W_2_ was composed of the ankle plantar flexors (MG, LG, and SOL). This muscle synergy was activated during the late stance phase to generate forward propulsion. The weightings and activations of the W_2_ were invariant across changes in walking speed. The W_3_ included the dominant hip flexor muscle (RF), the hip adductor muscle (AL), and the trunk stabilizer (ERE). The W_3_ was activated during swing initiation. The W_4_ was composed of the ankle dorsiflexor (TA) and the hip flexor muscle (RF, TFL) during the swing phase. The W_4_ was reduced at 2.0–4.0 km/h. The W_5_ was composed of the ankle dorsiflexor (TA) during the late swing phase.

### Shifting the CoA

**Figure 6:**
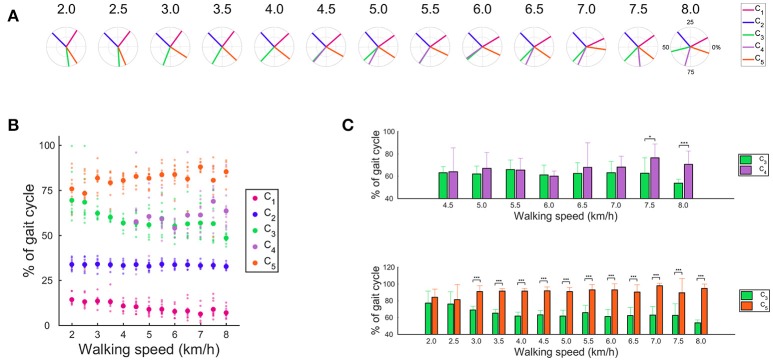
The CoAs in the muscle synergy. **(A)** Averaged CoA among subjects across walking speeds. Averaged CoA located in the polar coordinate, and the polar direction denotes the relative time over the gait cycle (time progresses counter-clockwise). **(B)** Averaged CoA and all subjects' CoA across walking speed conditions. Large dots denote the averaged CoA, and small dots indicate the CoA of each subject. **(C)** Difference in the CoA at each walking speed condition. Difference in the CoA between the C_3_ and C_4_ (upper) and between the C_3_ and C_5_ (below). Significance levels are illustrated as asterisks (*p < 0.05, ^***^p < 0.01).

The CoAs are indicated in Figures 6A,B. The CoA that was related to body support (C_1_) and the activation that contributed to swing initiation (C_3_) shifted to the earlier phase as the walking speed increased, whereas the CoA that was recruited during the swing phase (C_4_) and the activation that contributed to leg deceleration (C_5_) shifted to the later phase. Because the activations that produced forward propulsion (C_2_) were invariant, the CoA of the C_2_ was also invariant with changes in the walking speed. These main findings indicate that the CoA that contributed to body support (C_1_), swing initiation (C_3_), swing phase (C_4_) and leg deceleration (C_5_) shifted as the walking speed changed. We observed no significant difference in the CoA between the C_3_ and the C_5_ at 2.0 and 2.5 km/h (Figure 6C). The CoA of the C_3_ and the C_5_ separately located from 3.0 to 8.0 km/h (*p* < 0.01). The number of muscle synergies was 5 from 4.5 to 8.0 km/h, and the CoA of the C_3_ was close to the CoA of the C_4_ from 4.5 to 7.0 km/h. The CoA of the C_3_ were significantly earlier than the CoA of the C_4_ at 7.5 km/h and 8.0 km/h (*p* < 0.05).

### Joint angle, angular velocity, and angular accelerations

**Figure 7:**
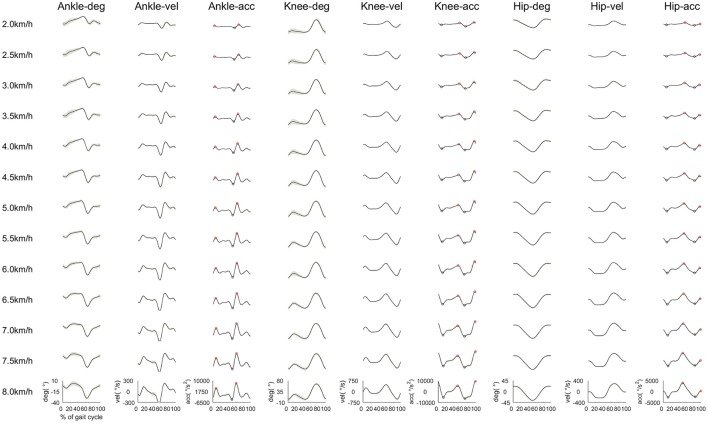
Averaged time series of the joint angle, velocity and acceleration in the ankle, knee and hip joints among all subjects. Black lines indicate the averaged joint angles, velocities, and accelerations. The gray shaded area is the standard deviation. The red open circles indicate peak accelerations of ankle dorsiflexion, knee flexion and hip flexion, respectively. The blue open circles denote peak accelerations of ankle plantar flexion, knee extension and hip extension, respectively. Each joint angle was set to 0° at the anatomical position.

To observe any changes in kinematic properties among walking speeds, we calculated the joint angle, angular velocity, angular acceleration, and variability of peak acceleration. We expected to observe a change in joint kinematics by shifting the CoA. The joint angles, angular velocities and angular accelerations among all walking speeds are shown in Figure 7. We observed that joint angles, angular velocities and angular accelerations were influenced by changes in walking speed. The joint angles, angular velocities and angular accelerations of ankle plantar flexion at approximately 60% of the gait cycle increased as the walking speed increased to generate strong propulsion. The angular velocities and angular accelerations of ankle dorsiflexion during the swing phase increased as the walking speed increased to move the plantar flexion position to dorsiflexion quickly. Knee joints were almost straight during the heel contact phase at slow walking speeds. However, the knee joints slightly flexed as the walking speed increased to absorb any shock during the heel contact phase. During the swing initiation, angular velocities, and angular accelerations of knee flexion increased when the walking speed increased. During the mid-swing phase, angular velocities and angular accelerations of knee extension increased, and angular accelerations of knee flexion increased just before the heel contact. The joint angles and velocities of hip extension during the propulsion phase increased as the walking speed increased to extend the stride length. To reduce the stride time, angular velocities of hip flexion from the initial swing phase to the mid-swing phase increased when the walking speed increased. The angular accelerations of hip extension before the heel contact increased to decelerate the swing leg.

### The peak angular accelerations

**Figure 8:**
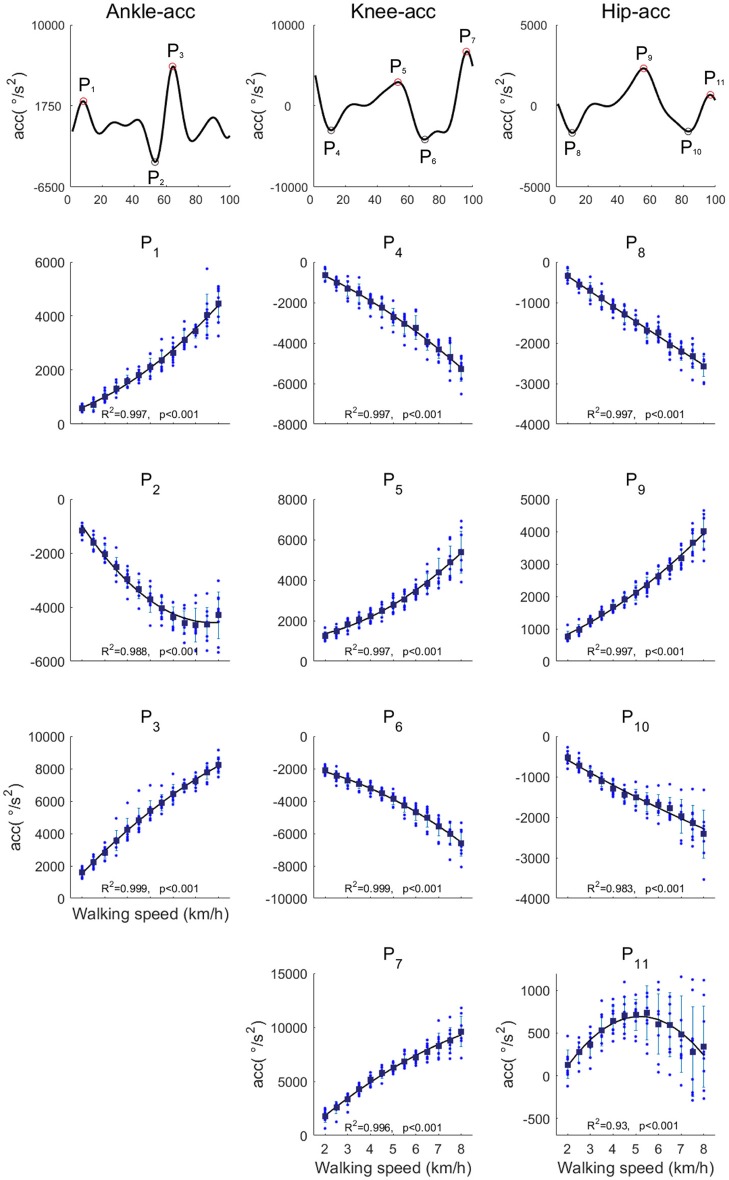
The peak angular accelerations in the ankle, knee and hip joints. The black-filled markers represent the averaged peak accelerations among all subjects. The black line is the quadratic regression curve.

We analyzed the peak angular accelerations among walking speeds (Figure 8). The peak angular acceleration timings, P_1_-P_11_, were the main peaks within the stance phase, pre-swing phase and late-swing phase at each joint. An example of peak angular acceleration timings is illustrated in Figure 8. The averaged peak angular acceleration timings (P_1_-P_11_) were 7.3 ± 1.48, 5.4 ± 3.65, 5.4 ± 2.03, 10.8 ± 1.74, 54.0 ± 1.89, 95.8 ± 2.34, 96.4 ± 1.29, 10.2 ± 1.44, 5.2 ± 2.05, 7.4 ± 1.09, and 97.4 ± 1.09% of the gait cycle, respectively. The fluctuations of peak angular acceleration timings were small among walking speeds. Because joint angles mostly change in the swing phase, maximum angular accelerations were exerted in the swing phase. Almost all of the peak angular accelerations increased as the walking speed increased, except angular acceleration of hip flexion just before the heel contact phase, which was the largest at approximately 5.5 km/h.

## Discussion

The purpose of this study was to identify the mechanism of control of walking speed based on the CoAs in different muscle synergies. We observed that the CoAs that contributed to body support (C_1_), swing initiation (C_3_), swing phase (C_4_) and leg deceleration (C_5_) shifted as the walking speed changed. The CoA of the C_1_ and the C_3_ shifted to the earlier phase as walking speed increased, whereas the CoA of the C_4_ and the C_5_ shifted to the later phase. The CNS might control walking speed by flexibly shifting the CoA in each muscle synergy.

### Shifting the CoA at fast walking speeds

We observed that the CoA shifted as walking speeds changed (Figure 6). The CoA that was related to body support (C_1_) shifted during the early phase as walking speed increased. As walking speed increased, the amplitudes of the ground reaction force also increased (Stansfield et al., 2001). Therefore, a large loading response is required for fast walking speeds. The CNS might shift the CoA that was related to body support (C_1_) to achieve this large loading response at fast walking speeds.

Both the C_3_ and C_4_ related to the swing leg. The CoAs of the C_3_ and C_4_ located in similar timing that corresponded to the initial-swing phase from 4.5 to 7.0 km/h. However, the CoA of the C_3_ located in the pre-swing phase, and the CoA of the C_4_ located in the mid-swing phase at 7.5 and 8.0 km/h, respectively. This result indicates that the CNS intensively controls muscle synergies within the swing phase at 7.5 and 8.0 km/h. The cost for the swing leg is considered small during normal walking speeds. For example, it has been reported that the net metabolic cost is 10% for the swing leg (Gottschall and Kram, 2005). In addition, the muscular effort for the swing leg was ignored in a mathematical model, or only considered during the swing initiation and the late-swing phase (Mochon and McMahon, 1980; Srinivasan and Ruina, 2006). However, contributions of the swing leg increase at fast walking speeds, and the metabolic work of the swing leg increases at high frequencies (Doke et al., 2005). This increase in the swing cost might occur through intense activation of the ankle dorsiflexor and hip flexor muscles in the swing leg (Figure 4). This intensive muscle activation for the swing leg during fast walking speeds might relate to shifting the CoA of the C_3_ and the C_4_. In addition, shifting the CoA of the C_3_ and the C_4_ at fast walking speeds might relate to a walk-to-run transition. The walk-to-run transition frequently occurs at approximately 7.0 km/h, and it has been suggested that increasing the swing cost is one of the factors inducing a gait transition (Prilutsky and Gregor, 2001; Pires et al., 2014). Specifically, ankle dorsiflexion power and activation of the rectus femoris during the swing phase of walking is higher than that of running when treadmill speeds are above the gait transition speed (Prilutsky and Gregor, 2001; Pires et al., 2014). In addition, specific peak activations in the muscle synergies change before and after the walk-to-run transitions (Hagio et al., 2015). The CoAs of the C_3_ and the C_4_ were significantly different at 7.5 and 8.0 km/h, which might be related to exceeding the walk-to-run transition speed. The CoA of the C_4_ is located during the mid-swing phase at 7.5 and 8.0 km/h. This finding indicates that accelerations of the swing leg are controlled during the mid-swing phase at 7.5 and 8.0 km/h. Because the swing cost during the mid-swing phase is considered small for normal walking speeds (Mochon and McMahon, 1980), the muscle activation during the mid-swing phase for fast walking speeds might relate to an inefficient swing leg. Therefore, shifting the CoA of the C_4_ might be associated with an inefficient swing leg.

### Shifting the CoA at slow walking speeds

Kinematic outputs are different between slow and normal walking speeds. It has been suggested that the control of extremely slow walking speeds demands postural stability rather than a swing leg (den Otter et al., 2004). In addition, Murray et al. (1984) reported that a peak burst of Gmed activity in the mid- or late-stance phase at slow walking speeds was larger than at fast walking speeds. We also observed this large activation of Gmed in the mid- or late-stance phase at slow walking speeds (Figure 4). Because the loading response was small, the activation of Gmed in the heel contact phase was small at slow walking speeds. The CNS may intensively control the single support phase at slow walking speeds. We observed that the CoA that contributed to body support (C_1_) occurred in the mid-swing phase at slow walking speeds. The W_1_ was mainly composed of knee flexor, hip adductor and hip extensor muscles (VL, Gmed, Gmax) to achieve a loading response and body support during the stance phase. At slow walking speeds, the CNS might intensively control the mid-stance phase by locating the CoA of the C_1_ in a mid-stance phase.

We observed that the CoA of the C_3_ and the C_5_ located in the swing initiation and the late-swing phases at normal and fast walking speeds, respectively. The C_3_ contributed to the swing leg, and the C_5_ was mainly activated just before the heel contact phase for ankle dorsiflexion. However, the CoAs of the C_3_ and the C_5_ concentrated on the mid-swing phase from 2.0 and 2.5 km/h. We observed that accelerations of hip flexion during the swing initiation at slow walking speeds were lower than at normal walking speeds. In addition, the ankle dorsiflexion just before heel contact was small (Figures 7, 8). At slow walking speeds, it is not necessary to accelerate the swing leg and large ankle dorsiflexion. Therefore, the function of the C_3_ and the C_5_ might be different between slow and normal walking speeds. We suggested that the C_3_ and the C_5_ contribute to foot clearance in the mid-swing phase at slow walking speeds. Shifting the CoAs of C_3_ and C_5_ might reflect alteration of control for slow walking speeds in the CNS.

### Clinical implications and comparison of muscle synergies between our extracted muscle synergies and previous studies

We determined the number of synergies based on the VAF, and we extracted 4 or 5 muscle synergies at each walking speed (Figures 2, 3, 5). Although we found slight differences across the composition of muscle synergies and the analyzed number of gait cycles, the number of synergies and functions of muscle synergies were similar to muscle synergies that were extracted in previous studies (Ivanenko et al., 2004; Neptune et al., 2009; Chvatal and Ting, 2013). Therefore, shifting the CoA as the walking speed changes has been observed in cases of other data sets of walking. Although we recruited 10 healthy men and fluctuation of the CoA among subjects was observed (Figure 6B), the shift of averaged CoAs among walking speeds were relatively consistent. Hence, we considered that the small number of subjects did not affect our results.

Because we selected only young healthy men as subjects, we need to remark on the possibility that the muscle synergy compositions and activations could be influenced by gender differences. Although it has been proposed that there are gender differences of kinematics during walking or other locomotion (von Tscharner and Goepfert, 2003; Zazulak et al., 2005; Chumanov et al., 2008), activation timings of muscles are similar between men and women. Therefore, we expect that shifting the CoA does not change between genders. However, the CoA during walking might be different between young and elderly adults. Monaco et al. (2010) demonstrated there was a slight time lag in the activation between young and elderly adults. Therefore, we suggest that shifting the CoA might be different between young and elderly adults.

In the introduction of this study, we remarked that muscle synergies might reflect disorders in the CNS (Clark et al., 2010; Rodriguez et al., 2013; Danner et al., 2015; Steele et al., 2015); however, clinicians may need to consider any observed difference of muscle synergies derived from kinematics of walking or disorders in the CNS. We revealed that the walking speed affected the activation of muscle synergies by investigating the CoA in healthy young adults. This finding indicates that muscle synergies depend on the walking speed. Therefore, the clinicians may need to consider effects of the walking speed when evaluating muscle synergies. We expect that this study can be applied in clinical fields to investigate the effect of the walking speed.

## Conclusion

We investigated altering the intensive activation of muscle synergies among various walking speeds to identify the mechanism of control of walking speed. We revealed that the CoA that contributed to body support (C_1_), swing initiation (C_3_), swing phase (C_4_) and leg deceleration (C_5_) shifted as the walking speed changed. This shifting of the CoA indicates that the CNS controls the intensive activation of muscle synergies during the regulation of walking speed. In addition, shifting the CoA might be associated with changes in kinematics or kinetics depending on the walking speed. We concluded that the CNS flexibly controls the activation of muscle synergies in the regulation of walking speed.

## Author contributions

BK, SH, TM, and MK, Conception and design of the experiments; BK and SH, Collection, analysis and interpretation of the data; BK, SH, and MK, Drafting the article or critically revising for important intellectual content; BK, SH, TM, and MK, Final approval of the version to be published.

### Conflict of interest statement

The authors declare that the research was conducted in the absence of any commercial or financial relationships that could be construed as a potential conflict of interest.
